# Development of a Novel NGS Methodology for Ultrasensitive Circulating Tumor DNA Detection as a Tool for Early-Stage Breast Cancer Diagnosis

**DOI:** 10.3390/ijms24010146

**Published:** 2022-12-21

**Authors:** Begoña Jiménez-Rodríguez, Alfonso Alba-Bernal, Esperanza López-López, María Elena Quirós-Ortega, Guillermo Carbajosa, Alicia Garrido-Aranda, Martina Álvarez, Ana Godoy-Ortiz, María Isabel Queipo-Ortuño, Luis Vicioso, Gema Díaz-Córdoba, María Dunia Roldán-Díaz, Jesús Velasco-Suelto, Cristina Hernando, Begoña Bermejo, Ana Julve-Parreño, Ana Lluch, Javier Pascual, Iñaki Comino-Méndez, Emilio Alba

**Affiliations:** 1 Unidad de Gestión Clínica Intercentros de Oncología Médica, Hospitales Universitarios Regional y Virgen de la Victoria, 29010 Malaga, Spain; 2 The Biomedical Research Institute of Málaga (IBIMA-CIMES-UMA), 29010 Malaga, Spain; 3 Centro de Investigación Biomédica en Red de Cáncer (CIBERONC-CB16/12/00481), 28029 Madrid, Spain; 4 Andalusia-Roche Network in Precision Medical Oncology, 41092 Sevilla, Spain; 5 Department of Surgical Specialties, Biochemical and Immunology, Faculty of Medicine, University of Málaga, 29071 Malaga, Spain; 6 Faculty of Medicine, University of Málaga, 29010 Malaga, Spain; 7 Histopathology Department, Hospital Clínico Universitario Virgen de la Victoria de Malaga, 29010 Malaga, Spain; 8 Radiology Department, Hospital Clínico Universitario Virgen de la Victoria de Málaga, 29010 Malaga, Spain; 9 Hospital Clínico Universitario de Valencia, Instituto de Investigación Sanitaria INCLIVA, 46010 Valencia, Spain

**Keywords:** circulating tumor DNA, ultra-deep sequencing, early breast cancer, liquid biopsy

## Abstract

Breast cancer (BC) is the most prevalent cancer in women. While usually detected when localized, invasive procedures are still required for diagnosis. Herein, we developed a novel ultrasensitive pipeline to detect circulating tumor DNA (ctDNA) in a series of 75 plasma samples from localized BC patients prior to any medical intervention. We first performed a tumor-informed analysis to correlate the mutations found in tumor tissue and plasma. Disregarding the tumor data next, we developed an approach to detect tumor mutations in plasma. We observed a mutation concordance between the tumor and plasma of 29.50% with a sensitivity down to 0.03% in mutant variant allele frequency (VAF). We detected mutations in 33.78% of the samples, identifying eight patients with plasma-only mutations. Altogether, we determined a specificity of 86.36% and a positive predictive value of 88.46% for BC detection. We demonstrated an association between higher ctDNA median VAF and higher tumor grade, multiple plasma mutations with a likelihood of relapse and more frequent *TP53* plasma mutations in hormone receptor-negative tumors. Overall, we have developed a unique ultra-sensitive sequencing workflow with a technology not previously employed in early BC, paving the way for its application in BC screening.

## 1. Introduction

Breast cancer (BC) is the most commonly diagnosed cancer in women worldwide (the Global Cancer Observatory, 2020). It is normally detected at early stages mainly due to surveillance programs employing mammograms in asymptomatic women aged between 40–50 to 70. Conversely, if the disease has spread to other organs outside the breast and axillary lymph nodes, it is largely incurable with current therapeutic options. BC is, in fact, the leading cause of cancer deaths among women (the Global Cancer Observatory, 2020). Once an abnormal finding is detected in a mammogram, a biopsy of the lesion remains the gold standard to confirm the presence of cancer cells. However, this well-established invasive clinical method imposes inherent risks on the patients such as breast bruising, swelling, infections and altered breast appearance. Moreover, it is well known that spatial heterogeneity is a common feature in cancer [1], and thus a localized solid biopsy, which only takes a small piece of the lesion for analysis, might not reflect the entire molecular landscape of the tumor.

Over the last decades, liquid biopsy has revolutionized the molecular oncology field as a non-invasive procedure to obtain crucial information from the tumor. It is a clinically validated methodology to detect minimal residual disease, treatment resistance and/or to serve as cancer treatment guidance, easily permitting continuous monitoring, and theoretically capturing molecular heterogeneity of the tumor [2,3,4]. Importantly, it represents a promising tool for early-stage diagnosis [5] and potentially for the screening of asymptomatic individuals for the presence of tumors. In this regard, little has been published about liquid biopsy in the screening process to detect BC in high-risk women. Several studies have been able to detect circulating tumor DNA (ctDNA) in the pre-treatment blood of BC patients with different sensitivities [3,6,7]; however, all of them required previous solid tumor genetic information to find cancer mutations in blood. In this regard, a seminal study developed a pan-cancer methodology to screen tumors through ctDNA detection and protein biomarkers without prior somatic analysis, but the sensitivity to detect BC was the lowest amongst all tumor types [8]. Considering all of the above-mentioned, it is crucial to find novel approaches to improve ctDNA detection in the first stages of cancer development and to demonstrate the utility of the liquid biopsy to detect BC in women with a high probability of presenting this disease.

**Figure 1:**
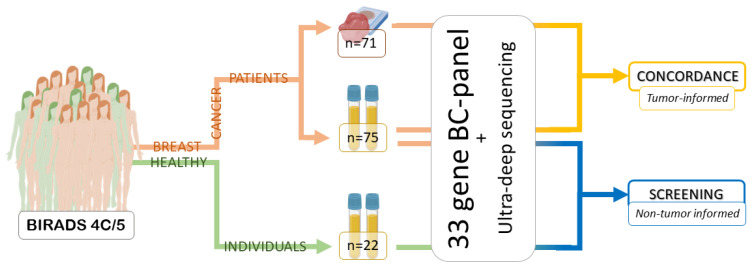
Workflow of the study. Patients with suspicious mammogram results (BIRADS 4C/5) were recruited and blood samples extracted prior to any medical procedure, together with fresh-frozen diagnostic tumor biopsies. Women with negative biopsies for BC were used as controls for the study. Then, a custom BC capture panel and ultra-deep sequencing were employed to analyze for concordance between tumor and plasma as well as to perform a non-tumor informed analysis.

In this study, we developed a novel method employing a custom BC capture sequencing panel with unique molecular identifiers (UMIs), ultra-deep sequencing and a custom bioinformatic pipeline, to detect tumor mutations in plasma from localized BC patients before diagnosis. We investigated the concordance between the mutational landscape of tumor and plasma and performed a non-tumor informed analysis to discriminate between cancer patients and healthy individuals that could potentially be used to non-invasively detect BC prior to any other medical intervention (Figure 1).

## 2. Results

### 2.1. The Genetic Landscape in Tumors and ctDNA of Localized BC Patients

A total of 75 early-stage BC patients were recruited for the study after obtaining a suspicious mammogram result (BIRADS 4C/5). For all of them, a blood sample was taken prior to any medical intervention. In 71 cases, a diagnostic pre-treatment core needle solid biopsy was also available. These BC patients were recruited between 2016 to 2018 and continue nowadays in clinical follow-up, with a median clinical follow-up of 4.36 years (Table 1).

A custom capture panel composed of the exonic regions of 33 genes involved in BC pathogenesis (see Section 4) was employed to characterize the mutational landscape of 71 pre-treatment solid biopsies and 75 plasma samples from the corresponding patients taken before any procedure; 4 of them were plasma-only samples and 1 tumor sample without the corresponding plasma. Firstly, the tumor DNA (n = 71) was sequenced using the Agilent SureSelect^XT HS^ technology, following protocol recommendations as previously reported [9]. Tumor sequencing was performed at 15,483X median coverage (Figure S1). Posterior bioinformatic processing utilizing UMIs to minimize sequencing errors provided a final median coverage of 1698X (Figure S1). Amongst the captured regions, only three were covered with less than 100X in more than 10% of the sequenced bases (Table S1). Amongst these regions, only one presented mutations in the TCGA BC database in 0.09 and 0.27% of the total samples (Table S1). In addition, all genes presented homogeneous coverage across samples (Figure S2). Next, a custom filtering was performed using information from public genomic databases to identify somatic mutations (see Section 4). Overall, 61 mutations were identified in 40/71 (56.33%) of the tumor samples. Amongst them, 33 were located in the *PIK3CA* gene (54.09%), 12 in *TP53* (19.67%) and 4 in *GATA3* (6.55%) (Table 2 and Table S2; Figure S3), representing the most frequently mutated genes in our tumor set.

**Figure 2:**
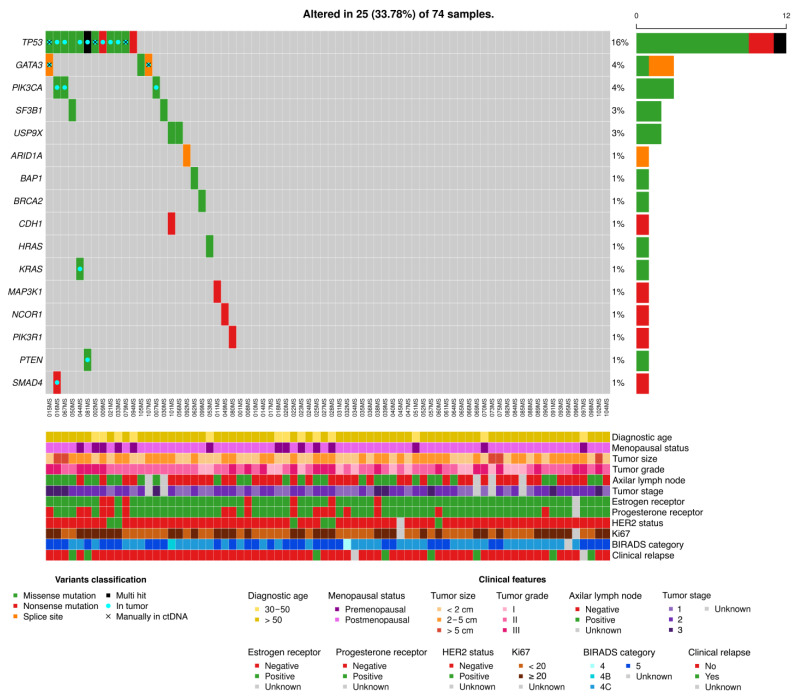
Somatic mutations observed in the plasma samples of localized BC patients. Top part. Mutations identified in the plasma samples both in the tumor and non-tumor informed sub-studies. Bottom part. Clinicopathological characteristics of the included patients showing the current clinical status (clinical relapse row).

To investigate the concordance between the mutations found in tumors and in plasma, the custom capture panel was also applied to plasma DNA (n = 75). Plasma sequencing reached 17,704X median coverage (Figure S1). In total, 74 plasma samples from the patients were sequenced, 4 of them without tumor tissue available and one plasma sample failing in the sequencing process. After UMIs processing, the median coverage was 2525X (Figure S1). Amongst the sequenced gene regions, three presented low coverage and all genes showed homogeneous coverage (Figure S2, Table S1). Amongst these low-coverage regions, mutations were observed in two of them in the TCGA BC database, identified in 0.09% and 0.27% of the total samples (Table S1). After bioinformatic analyses using the established mutation caller (see Section 4), 13/61 (21.31%) tumor mutations were found in plasma that were also present in the corresponding tumors; 7 mutations in the *TP53* gene (53.84%) and 3 in *PIK3CA* (23.07%) as the most frequently mutated genes (Figure 2 and Figure S4; Table 2 and Table S3).

Additionally, all mutations previously identified in tumors were manually inspected in the plasma sequencing raw data. Aligned data were used to identify supporting reads for the variant alleles using the IGV software v2.15.2 (see Section 4). Mutations found in at least two reads with different genomic coordinates passed to the next analysis step as previously recommended [9]. To consider the variants as valid, a Fisher’s exact test was applied using sequencing data from 22 plasma healthy controls and non-mutated patients’ plasma samples (Table S4). To perform the statistic tests, absolute allele counts for the variants and wild-type alleles were calculated both in the corresponding plasma sample, in 22 healthy plasma controls and plasma samples from BC patients negative for each specific mutation (Table S4). When considering only the plasma samples from healthy controls, five mutations from four different patients were rescued from plasma sequencing using manual inspection (see Section 4, Table 2 and Tables S3 and S4). In contrast, including plasma samples from BC patients in the statistic tests introduced a certain degree of noise and the mutation c.742C>T in sample 079MS was not detected despite being close to significance (*p*-value = 0.053) (Table S4). Considering Fisher’s tests calculated using pure plasma controls, three mutations were located in the *TP53* gene and two in *GATA3*. Interestingly, the two structural variants in *GATA3* with robust sequencing stats recovered using manual inspection evidences the difficulties some callers have to identify indels. Considering the detected variants both by the caller and by the manual inspection, 18/61 (29.50%) somatic variants found in tumor tissue were also discovered in plasma samples (Figure 2 and Figure S4; Table 2 and Table S3).

### 2.2. Panel Utility for BC Detection Using a Non-Tumor Informed Pipeline and Association with Clinicopathological Variables

To investigate the capacity of our next generation sequencing (NGS) pipeline to be used to non-invasively detect BC after suspicious mammograms, a bioinformatic non-tumor informed analysis was developed. In this analysis, the somatic mutations’ information from solid biopsies was disregarded and only Mutect2 was employed to detect mutations in plasma samples using 1 UMIs families and no filters in variant allele frequencies (VAFs) (see Section 4). Variants were considered as shed by the tumor if (i) they affected exonic regions, (ii) were annotated in the COSMIC, TCGA BC and TCGA databases including all cancer types as well as if (iii) there were variant-supporting reads aligned in two or more different genomic coordinates manually visualized using the IGV software. Following the mentioned criteria, 25/74 (33.78%) individuals presented tumor mutations detected in their plasma (Figure 2), 16 of the mutations were not observed in the previous tumor-informed analysis (Table 3 and Table S5). Amongst them, a new mutation was observed in the *TP53* gene in the sample 081MS, different to the one detected in the tumor sequencing (Table 2, Table 3 and Table S5; Figure 2). Additionally, ctDNA mutations were found in eight plasma samples in whose corresponding tumor biopsies no mutations were detected (Table 3 and Table S5; Figure 2). Finally, a mutation was found in one plasma sample with no tumor tissue available (Table 3 and Table S5). Overall, amongst the 25 different plasma mutations, *TP53* (13 mutations, 52%), *PIK3CA* (3 mutations, 12%) and *GATA3* (3 mutations, 12%) were the most frequently affected genes (Table 2, Table 3 and Table S5).

Then, 22 plasma samples from healthy individuals were sequenced with the same sequencing panel, protocol conditions and coverage as the plasma samples from patients (Wilcoxon test *p*-value = 0.7112) (Figures S5 and S6). After applying the same bioinformatic pipeline as for BC cases, mutations were found in the plasma of 3/22 (13.63%) controls (see Section 4; Figure S5). One mutation affected the *MAP3K1* gene (p.N1125D), which was described in the COSMIC database in one breast cancer tumor sample, one mutation was located in the *ERBB2* gene (p.V842I), which has been observed to be substantially more frequent in colon and endometrial cancers, and an additional one was found in the *SMAD4* gene (p.R361H), which is also remarkably frequent in colon adenocarcinoma and pancreatic cancer (Table S6).

Considering our findings, the employment of the custom capture panel together with an ultra-deep sequencing and a custom non-tumor informed bioinformatic analysis led to a sensitivity of 31.08% (95% CI: 20.83% to 42.90%), a specificity of 86.36% (95% CI: 65.09% to 97.09%) and a positive predictive value (PPV) of 88.46% (95% CI: 71.75% to 95.86%) for breast cancer detection in our cohort. Importantly, the calculated sensitivity increases with the disease stage, from 21.43% (95% CI 8.30% to 40.95%) in stage 1 to 22.45% (11.77% to 36.62%) in stage 2 and 44.44% (95% CI: 13.70% to 78.80%) in stage 3.

The association of clinicopathological variables with mutation detection in plasma were also investigated. In detail, the ctDNA positivity in plasma, the mutations’ median VAF, the number of mutations per sample as well as samples with mutations in *TP53* were studied for their association with clinical characteristics (Table S7). Overall, the higher median VAF was associated with higher tumor grade (*p* = 0.0463), the presence of more than one plasma mutation in plasma with the likelihood of clinical relapse (*p* = 0.0237) and *TP53* mutations in plasma more frequently observed in hormone receptor (HR)-negative tumors (estrogen receptor (ER)-negative *p* = 0.0316; progesterone (PR)-negative, *p* = 0.0257). Additionally, the association of clinical relapse and plasma mutations with high median VAF, defined as mutations with >0.05% in AF, was interestingly close to significance (*p* = 0.059) (Table S7). To note, 38.35% of the patients included herein were asymptomatic and diagnosed by the BC early detection program. Amongst them, 28.57% of them presented plasma mutations, a similar percentage as the 33.33% of symptomatic women with mutations.

## 3. Discussion

In this study, we described the utility of a novel custom capture panel used together with ultra-deep sequencing to detect ctDNA in pre-treatment plasma samples from localized BC patients. We aimed to (i) study the correlation of detected variants between tumor tissue and plasma and (ii) the panel efficacy to detect ctDNA as biomarker for BC in non-diagnosed patients. To our knowledge, this is the first time that a similar technology has been employed in plasma samples from early BC patients, both to correlate genetic landscapes between tumor and plasma as well as to detect BC in women with suspicious mammograms. Previous studies have tried to use the amplification NGS technologies alone [6] or in combination with other blood-circulating components with limited results in BC [8]. In addition, the methodology used herein has demonstrated its capability to detect minute amounts of mutant DNA, although it had been never employed in plasma samples from localized cancers to date [9].

Firstly, we performed ultra-deep sequencing in tumor DNA and the corresponding plasma to correlate the mutational landscape. In tumor sequencing, we observed the genes *TP53*, *PIK3CA* and *GATA3* as the more frequently mutated ones, an observation in line with findings in previous in studies [10,11] and databases (TCGA for BC). Regarding their biological meaning, *TP53* is a gene encoding the p53 protein, which is involved in gene transcription initiation with a role in cell cycle arrest, cellular senescence and apoptosis, amongst others. Then, p53 disruption leads to cell homoeostasis dysregulation at several levels altering cell fate [12]. On the other hand, the *PIK3CA* gene encodes the catalytic subunit of PI3K and the alteration of this protein provokes the dysregulation of signaling pathways involved in cell survival, apoptosis, proliferation, motility and adhesion [13]. Finally, the GATA3 gene encodes for a transcriptional regulator. It has been demonstrated that breast cells with impaired expression of the gene are poorly differentiated, leading to metastatic progression [14].

When comparing mutations in tumor tissue and plasma, we observed a concordance of 29.50%. Previous studies have demonstrated similar results using amplification methodologies but studying a remarkably smaller number of genes, limiting the tumor genetic information inferred from them [15]. In addition, we have developed a custom bioinformatic pipeline to detect ctDNA mutations in plasma missed by an automatic variant caller. The same technology and sequencing depth have been tested previously, demonstrating a robust variant identification around a VAF of 0.15% and less efficient detection of variants down to 0.075% [9]. Herein, we increased the detection sensitivity by identifying variants below 0.075% using a different sequencing platform and a custom bioinformatic pipeline (Table 2, Section 4). Importantly, we also found mutations in eight plasma samples, whose corresponding tumors bore no detectable mutations (Table 3 and Table S5). This observation highlights the tumor heterogeneity as well as the commonly mentioned liquid biopsy’s capacity to provide a more complete tumor genetic landscape as compared to solid biopsy, which is limited by the tumor tissue captured by core needles [16,17].

In addition, we explored the panel clinical validity in detecting BC in women with suspicious BIRADs 4c and five lesions in the mammograms. We developed a non-tumor informed pipeline using the plasma DNA sequencing of our series of patients as well as 22 plasma samples from women who enrolled into the study with suspicious mammograms but were eventually not diagnosed with BC. We could observe high specificity (86.36%) but relatively low sensitivity (31.08%) in identifying individuals affected by BC. These findings highlight the difficulties in detecting ctDNA in localized BC even in pre-treatment blood samples with a demonstrated limit of detection down to 3 mutant molecules in 10,000 wild-type (Table 2). Concordant results were reported in other studies utilizing different technologies such as the droplet-digital PCR [3,18,19]. However, the high specificity observed using our methodology with a remarkably high PPV of 88.46% remains noteworthy. To note, the sensitivity of our methodology increases with the disease stage from 21.43% in stage 1 to 44.44% in stage 3, an observation previously reported for cancer detection using plasma DNA sequencing [20]. Another study demonstrated the possibility of detecting ctDNA in localized BC at lower sensitivity but requiring tumor information to design patient-specific NGS panels [7]. Another recent study has tried to explore methodologies for BC screening using liquid biopsy and NGS panels together with UMIs. Importantly, tumor genetic information was also necessary therein to design patient-specific panels and the authors only detected ctDNA in 14.1% of the pre-treatment plasma samples from early BC patients [6]. Similarly, a seminal study investigated the utility of using a pan-cancer high-sensitive NGS technology, together with circulating biomarkers to early detect eight tumor types. Strikingly, the sensitivity to detect localized BC in this study was similar to the one demonstrated herein, but it required the addition of other circulating biomarkers such as proteins [8]. Moreover, it is important to highlight that the set of localized BC patients included in the mentioned study had a higher tumor grade than the ones studied here. This might have enhanced the probability of ctDNA detection in the plasma samples. Overall, patient-specific NGS panels have demonstrated more sensitivity in detecting minute amounts ctDNA but requiring previous tumor sequencing, and also increasing total costs for panel design and optimization [7]. The genes included in the NGS panel described herein comprises more than 83% of the mutated genes in BC (TCGA database). Therefore, increasing the number of genes in the panel would not boost ctDNA detection sensitivity but increase sequencing costs to achieve the necessary depth.

Importantly, we could test the association between mutations in plasma and multitude of patient’s clinicopathological characteristics (Table S7). We observed the statistical significance between higher median VAF and higher tumor grade and more frequent plasma *TP53* mutations in HR-negative tumors. To note, we could associate the presence of more than one mutation in plasma with the likelihood of clinical relapse, in part thanks to the long clinical follow-up of the patients included in this study. Moreover, it is also important to highlight the observation of a trend in the association between the median VAF with patients’ clinical relapse (Table S7). This is one of the first studies suggesting that the pretreatment plasma sequencing could provide information about the clinical outcome in localized BC patients. In addition, the median VAF of plasma variant was associated with the tumor stage, a finding that was previously demonstrated [15,21]. Moreover, the association between more frequent *TP53* mutations in HR-negative patients has been also previously demonstrated in plasma sequencing from BC cancer patients [22,23], where the authors pointed certain implications in response to anti-HER2 treatments. Further studies increasing the number of patients would provide more insights about the association of clinicopathological variables and patients’ clinical outcomes with plasma mutations.

## 4. Materials and Methods

### 4.1. Patients and Women with Negative Biopsies

Plasma samples from 75 women with BIRADS 4c/5 mammography findings were collected just before tissue biopsy prior to cancer diagnosis and treatment. The patients presented early BC disease at diagnosis, defined as local or locally advanced disease without the presence of metastases.

Tumor biopsies were extracted using core needle biopsies, which were fresh frozen. Immunohistochemical (IHC) analysis was performed to quantify the expression of human epidermal growth factor receptor 2 (HER2), hormone receptors (HR) and Ki67. The estrogen receptor (ER) and progesterone receptor (PR) were considered positive in tumors presenting more than 1% nuclear-stained cells. HER2 staining was scored according to guidelines [24]. HER2 status was considered positive when graded as 3+, while 0 to 1+ were negative and 2+ was an inconclusive result, and the silver in situ hybridization was performed. Tumor stages were defined as per clinical guidelines [25].

Plasma samples from women presenting negative biopsies for BC included in this study were used as controls. In detail, women with BIRADS 4C and negative biopsies were subjected to mammography 6 months after the first test. If there is absence of disease, these women enter into the normal BC screening guidelines. On the other hand, women with BIRADS 5 were subjected to mammography after 6 months and 1 year from the first test. If there is absence of disease, these women were clinically followed up with on a 1-year/2-year basis depending on age or clinical status. All these women remain free-of-disease to date.

### 4.2. Blood Sample Processing

A total of 10 mL of plasma were obtained from each recruited individual in STRECK tubes (Streck, La Vista, NE, USA). Within 2 h after collection, plasma was isolated from whole blood by centrifugation for 10 min at 3000 rpm at room temperature and stored at −80 °C until the circulating-free DNA (cfDNA) extraction.

### 4.3. DNA Extraction and Quantification from Plasma and Solid Biopsies

cfDNA was extracted from plasma samples using the QIAamp Circulating Nucleic Acid Kit (Qiagen, Hilden, Germany) according to the manufacturer’s instructions. Tumor DNA was isolated from fresh frozen tissue samples using the DNeasy Blood and Tissue Kit (Qiagen, Hilden, Germany) following the manufacturer’s instructions. cfDNA and DNA from solid tumors were quantified using the droplet-digital PCR (Bio-Rad, Hercules, CA, USA) and the RNAseP assay (Thermo Fisher Scientific, Waltham, MA, USA), as previously published [18].

### 4.4. Sequencing BC Panel Design

The genes to be included in the custom panel were selected as follows: (i) Genes with mutations in BC in ≥1% of samples from a public database (https://www.cbioportal.org/, accessed on 10 January 2021), (ii) genes analyzed and mutated in BC samples from a seminal study [10], (iii) genes with interest in BC biogenesis and (iv) other interesting genes showing low mutation frequencies in BC databases but with important roles in other cancer types. Thus, the panel included the coding regions of the following gene list: *AKT1*, *ARID1A*, *ATM*, *BAP1*, *BRAF*, *BRCA1*, *BRCA2*, *CBFB*, *CDH1*, *CDKN1B*, *CTCF*, *ERBB2*, *ESR1*, *GATA3*, *HRAS*, *KDM6A*, *KRAS*, *MAP2K4*, *MAP3K1*, *MEN1*, *NCOR1*, *NF1*, *PBRM1*, *PIK3CA*, *PIK3R1*, *PTEN*, *RB1*, *RUNX1*, *SF3B1*, *SMAD4*, *TBX3*, *TP53*, *USP9X* (Table S8). The custom NGS panel for BC was designed using the SureDesign software (Agilent, Santa Clara, CA, USA) with the next settings: 5x for tiling, least stringent for masking, XTHSBoosting for Boosting and a value of 30 for extension into repeats.

### 4.5. Sequencing Library Preparation

SureSelect^XT HS^ (Agilent, Santa Clara, CA, USA) methodology was employed to generate sequencing libraries. We constructed libraries using a median input plasma DNA of 39.78 ng (max 173.91 ng–min 5.01 ng) from BC patients and 21.78 ng (max 113.52 ng–min 1.71 ng) from healthy individuals and a median tissue DNA of 199.50 ng (max 200 ng–min 6.95 ng) from tumors. The DNA from tissue was fragmented using the SureSelect Enzymatic Fragmentation kit (Agilent, Santa Clara, CA, USA) and the libraries prepared using the SureSelect^XT^ Target Enrichment System kits (Agilent, Santa Clara, CA, USA) following the manufacturer’s indications. All PCR steps were carried out in the C1000 Touch Thermal Cycler (Bio-Rad, Hercules, CA, USA).

Fragment ranges from libraries were assayed with the Bioanalyzer High-Sensitivity DNA chips (Agilent, Santa Clara, CA, USA) and quantified using the KAPA Library Quantification Kit (Roche, Basel, Switzerland). For tumor tissue DNA sequencing, eight pools containing eight to nine library samples per pool were prepared and sequenced. For BC plasma DNA, 8 pools containing 9 to 10 library samples per pool and 3 pools containing 7 to 8 library samples per pool from healthy controls’ plasma DNA were also prepared and sequenced. A total of 19 lanes (1 lane per pool) were employed to sequence the libraries aiming to obtain ultra-deep sequencing of around 20,000X before de-duplication in the DNBseq-G400 platform (MGI, Hong Kong) at 100 pair-end reads following the manufacturer’s instructions for UMIs sequencing.

### 4.6. Sequencing Data Processing

We created a custom pipeline for the processing of the SureSelect^XT HS^ (Agilent, Santa Clara, CA, USA) sequencing data (Figure S7). We initially performed quality control of the sequencing data using fastQC v0.11.9. Next, we trimmed reads for adapters and quality filtered using trim-galore v0.6.7. To perform the processing steps that involve barcoded data, we used a subset of fgbio tools v1.5.1. We mapped the data to the GRCh38 reference genome using bwa v0.7.17. We next used fgbio GroupReadsByUmi to collapse by barcode using the Identity option to take into account that SureSelect^XT HS^ barcodes are degenerate. Next, we generated consensus reads using fgbio CallMolecularConsensusReads. The generated consensus reads were mapped again with bwa. We then filtered these aligned consensus reads using fgbio FilterConsensusReads, requiring a minimum base quality of 30 and keeping consensus reads supported by at least a minimum number of reads. We then used fgbio ClipBam to remove forward and reverse reads overlapping regions.

Finally, we performed variant calling with Mutect2 (gatk v4.2.2.0-1) including a panel of non-cancer DNA and a germline variant annotation file for the GRCh38 genome, obtained from the gatk resource bundle, that we used to annotate variants for filtering and only considering the regions included in the SureSelect panel. We annotated the variants with ANNOVAR [26] v20200608 with custom made databases for COSMIC version 95 and TCGA, downloading the calling results generated with the MuTect2 variant caller from the GDC data portal [27] for the latter.

### 4.7. Variant Filtration and Analysis

For tumor, we used a more stringent approach in order to create a solid reference to compare with the ctDNA findings. We generated consensus reads requiring a minimum of 3 contributing reads per read family. We accepted as valid calls only variants with VAF > 0.05 that were also present in either COSMIC or TCGA, increasing the VAF threshold to VAF > 0.2 for Formalin-Fixed Paraffin-Embedded tissues.

In the case of ctDNA, we identified mutations using two methods: (i) Stringent; using the same approach as described above but filtering for a minimum of 1 read per read family, with no VAF threshold applied. To consider mutations not found in the tumor as detected in plasma, we required them to have a duplex configuration, with at least two fragments mapping to different coordinates and to be present in both COSMIC and TCGA BC. We applied the same processing approach to control samples. (ii) Exploratory; visualizing the alignments in the IGV genome browser [28] in order to identify mutations previously found in the corresponding tumors but missed by variant callers. When we detected the presence of the variant not reported by the variant caller, we counted the number of reads carrying the mutation in a given sample and the number of reads for the wild-type allele. Then, we compared them against the same read proportions in controls and BC plasma samples without the corresponding mutation using a Fisher test (Table S4).

### 4.8. Statistical Analyses and Data Visualization

We performed statistical analyses and plotted data with R (https://www.R-project.org/, accessed on 1 October 2022). Fisher’s exact test or Chi-square test were applied when appropriate both for testing association between clinicopathological variables and plasma sequencing data, as well as in sequencing data analyses. Wilcoxon test was also applied to test for differences in sequencing coverage between cases and controls (Figure S6). The threshold for statistical significance was established at *p* < 0.05. Sensitivity, specificity and PPV values were calculated using the caret v6.0.93 package. The oncoplot function from the maftools [29] v2.12.0 package was used to plot mutations and clinicopathological data.

## 5. Conclusions

Considering the above discussed, our NGS plasma-only workflow showed enhanced capacities to detect ctDNA in localized BC patients at the very first diagnosis stages, improving detection sensitivity and adding evidence that ctDNA could help in the diagnostic process of asymptomatic population. This is supported by the similar percentages in plasma mutation identification between symptomatic and asymptomatic women of 33.33% and 28.57%, respectively. In this regard, we developed a custom bioinformatic pipeline to identify plasma mutations without tumor information, demonstrating high PPV and suggesting similar approaches could be tested as a screening tool for BC. We also demonstrated that by sequencing early BC patients’ plasma DNA, it is feasible to obtain important information about the disease as well as to predict the clinical outcome in these patients.

## Figures and Tables

**Table 1:** Clinicopathological characteristics of the localized/locally advanced BC patients included in the study.

Clinical Characteristics	n (%)
**Diagnostic age (years)**	
30–50	13 (17.6)
>50	61 (82.4)
**Tumor type**	
IDC	59 (79.7)
DCIS	5 (6.8)
ILC	3 (4.1)
PC	1 (1.4)
TC	3 (4.1)
MC	3 (4.1)
**Tumor size**	
<2 cm	32 (43.2)
2–5 cm	37 (50.0)
>5 cm	5 (6.8)
**Tumor grade**	
I	15 (20.3)
II	37 (50.0)
III	22 (29.7)
**Axilar lymph node**	
Positive	29 (39.1)
Negative	40 (54.0)
Unknown	5 (6.7)
**Disease stage**	
Stage 1A	19 (25.6)
Stage 1B	3 (4.0)
Stage 2A	28 (37.8)
Stage 2B	10 (13.5)
Stage 3A	5 (6.7)
Stage 3C	4 (5.4)
Unknown	5 (6.7)
**Estrogen receptor**	
Positive	66 (89.2)
Negative	7 (9.5)
Unknown	1 (1.4)
**Progesterone receptor**	
Positive	56 (75.7)
Negative	17 (23)
Unknown	1 (1.4)
**HER2 status**	
Positive	6 (8.1)
Negative	67 (90.5)
Unknown	1 (1.4)
**BIRADS category**	
4/B/C	40 (54.1)
5C	33 (44.6)
Unknown	1 (1.4)
**Clinical relapse**	
Yes	8 (10.8)
No	64 (86.5)
Unknown	2 (2.7)

IDC, invasive ductal carcinoma; ILC, invasive lobular carcinoma; DCIS, ductal carcinoma in situ; PC, papillary carcinoma; TC, tubular carcinoma; MC, mucinous carcinoma.

**Table 2:** Mutations detected in tumor and plasma samples.

Tumor					Plasma		
Sample	Gene	Nucleotide Change	Aa Change	VAF (%)	Caller Detected No (N)/Yes (Y)	Manually Detected No (N)/Yes (Y)	VAF (%)
001MS	*PIK3CA*	c.G3145C	p.G1049R	14.6	N	N	-
002MS	*CDH1*	c.C2245T	p.R749W	5.3	N	N	-
	*TP53*	c.G524A	p.R175H	54.5	N	Y	0.2
	*PIK3CA*	c.G1252A	p.E418K	35.0	N	N	-
	*PIK3CA*	c.A3140T	p.H1047L	34.4	N	N	-
007MS	*PIK3CA*	c.A3140G	p.H1047R	31.2	Y	N	0.4
009MS	*TP53*	c.C637T	p.R213X	45.2	Y	N	0.8
010MS	*PIK3CA*	c.A3140G	p.H1047R	15.0	N	N	-
014MS	*PIK3CA*	c.A3140G	p.H1047R	25.9	N	N	-
015MS	*GATA3*	c.922-3_922-2delCA	p.X308_splice	22.4	N	Y	0.08
	*TP53*	c.A377G	p.Y126C	58.2	N	Y	0.24
016MS	*TP53*	c.G743T	p.R248L	37.9	Y	N	4
	*SMAD4*	c.C725G	p.S242X	23.7	Y	N	3.2
	*PIK3CA*	c.A3140T	p.H1047L	35.1	Y	N	4.6
017MS	*PIK3CA*	c.A3140G	p.H1047R	27.3	N	N	-
021MS	*TP53*	c.376-2A>G	p.X126_splice	33.4	Y	N	1.8
022MS	*KDM6A*	c.C1747T	p.Q583X	12.5	N	N	-
023MS	*TP53*	c.G524A	p.R175H	63.0	N	N	-
	*PIK3CA*	c.G1633A	p.E545K	39.3	N	N	-
030MS	*GATA3*	c.922-3_922-2delCA	p.X308_splice	38.7	N	N	-
	*PIK3CA*	c.G1633A	p.E545K	77.0	N	N	-
031MS	*PIK3CA*	c.G1633A	p.E545K	15.7	N	N	-
032MS	*PIK3CA*	c.G353A	p.G118D	6.9	N	N	-
	*PIK3CA*	c.G2908A	p.E970K	14.7	N	N	-
	*PIK3CA*	c.A3140G	p.H1047R	11.4	N	N	-
	*PIK3CA*	c.A3140T	p.H1047L	3.0	N	N	-
033MS	*TP53*	c.A503T	p.H168L	37.6	Y	N	0.37
035MS	*PIK3CA*	c.G1633A	p.E545K	34.8	N	N	-
036MS	*NF1*	c.3478delG	p.G1160Vfs*6	5.5	N	N	-
	*PIK3CA*	c.A3140T	p.H1047L	31.5	N	N	-
039MS	*AKT1*	c.G49A	p.E17K	33.7	N	N	-
	*NCOR1*	c.G6751T	p.G2251C	10.3	N	N	-
040MS	*PIK3CA*	c.G1093A	p.E365K	21.7	N	N	-
	*PIK3CA*	c.G1624A	p.E542K	40.0	N	N	-
044MS	*KRAS*	c.G35C	p.G12A	29.3	Y	N	0.97
	*TP53*	c.G587C	p.R196P	51.2	Y	N	1.2
045MS	*AKT1*	c.G49A	p.E17K	6.9	N	N	-
047MS	*PIK3CA*	c.A3140G	p.H1047R	7.2	N	N	-
052MS	*PIK3CA*	c.A1637G	p.Q546R	19.8	N	N	-
	*PIK3CA*	c.A3073G	p.T1025A	21.6	N	N	-
056MS	*PIK3CA*	c.G1624A	p.E542K	17.9	N	N	-
057MS	*PIK3CA*	c.G1633A	p.E545K	32.1	N	N	-
060MS	*MAP3K1*	c.813_814del	p.R273Sfs*27	11.6	N	N	-
064MS	*PIK3CA*	c.T1035A	p.N345K	34.9	N	N	-
065MS	*GATA3*	c.922-3_922-2delCA	p.X308_splice	23.3	N	N	-
	*PIK3CA*	c.A3140G	p.H1047R	25.9	N	N	-
066MS	*ERBB2*	c.G2305T	p.D769Y	23.4	N	N	-
	*PIK3CA*	c.G1624A	p.E542K	27.5	N	N	-
067MS	*TP53*	c.A842C	p.D281A	51.8	Y	N	0.31
	*PIK3CA*	c.G3145C	p.G1049R	86.2	Y	N	0.32
079MS	*TP53*	c.C742T	p.R248W	9.1	N	Y	0.05
	*PIK3CA*	c.A1637G	p.Q546R	11.9	N	N	-
080MS	*PIK3CA*	c.G1633A	p.E545K	26.4	N	N	-
081MS	*PTEN*	c.T406C	p.C136R	54.0	Y	N	3.3
	*TP53*	c.G743A	p.R248Q	52.9	Y	N	1.5
093MS	*PIK3CA*	c.A1634G	p.E545G	29.1	N	N	-
095MS	*PIK3CA*	c.A3140T	p.H1047L	52.0	N	N	-
099MS	*PIK3CA*	c.A3140T	p.H1047L	28.6	N	N	-
101MS	*SF3B1*	c.A2098G	p.K700E	20.9	N	N	-
104MS	*TP53*	c.A715G	p.N239D	22.2	N	N	-
107MS	*GATA3*	c.922-3_922-2delCA	p.X308_splice	40.1	N	Y	0.03

Aa, aminoacid; VAF, variant allele frequency.

**Table 3:** Mutations detected exclusively in plasma samples. It is indicated whether the mutations are described in databases (COSMIC and TCGA) as well as whether the tumor biopsy was sequenced and any mutation was identified.

Sample	Gene	Nucleotide Change	Aa Change	VAF (%)	COSMIC + TCGA BC + TCGAall No (N)/Yes (Y)	Tumor Biopsy Sequenced No (N)/Yes (Y)	Any Mutation in Tumor No (N)/Yes (Y)
011MS	*MAP3K1*	c.C1292A	p.S431*	0.238	Y	Y	N
030MS	*SF3B1*	c.C1898T	p.A633V	0.389	Y	Y	Y
049MS	*NCOR1*	c.3022C>T	p.Q1008*	0.361	Y	Y	N
050MS	*SF3B1*	c.2098A>G	p.K700E	1.4	Y	Y	N
	*TP53*	c.733G>A	p.G245S	0.321	Y	Y	N
053MS	*HRAS*	c.34G>T	p.G12C	0.894	Y	Y	N
056MS	*USP9X*	c.1795C>T	p.R599C	0.481	Y	Y	Y
062MS	*BAP1*	c.709C>T	p.R237C	0.405	Y	Y	N
080MS	*PIK3R1*	c.1669C>T	p.R557*	0.318	Y	Y	Y
081MS	*TP53*	c.637C>T	p.R213*	0.643	Y	Y	Y
092MS	*ARID1A*	c.2879-1G>A	p.X960_splice	0.329	Y	Y	-
094MS	*TP53*	c.528C>A	p.C176*	0.327	Y	Y	N
099MS	*BRCA2*	c.1786G>A	p.D596N	0.450	Y	Y	Y
101MS	*USP9X*	c.1795C>T	p.R599C	0.209	Y	Y	Y
	*CDH1*	c.220C>T	p.R74*	0.279	Y	Y	Y
105MS	*GATA3*	c.914G>A	p.R305Q	0.277	Y	Y	N

Aa, aminoacid; VAF, variant allele frequency.

## Data Availability

The datasets used and/or analyzed during the current study are available from the corresponding author on reasonable request.

## Supplementary Materials

The supporting information can be downloaded at: https://www.mdpi.com/article/10.3390/ijms24010146/s1.
