# Supplementary material for: Development of a Novel NGS Methodology for Ultrasensitive Circulating Tumor DNA Detection as a Tool for Early-Stage Breast Cancer Diagnosis
Source: Int J Mol Sci. 2022 Dec 21;24(1):146. doi: 10.3390/ijms24010146 (PMC9820510; doi:10.3390/ijms24010146)
Supplement: Supplementary file 1 [file ijms-24-00146-s001.zip › Supplementary Figures_S1-S7_revision.pdf]

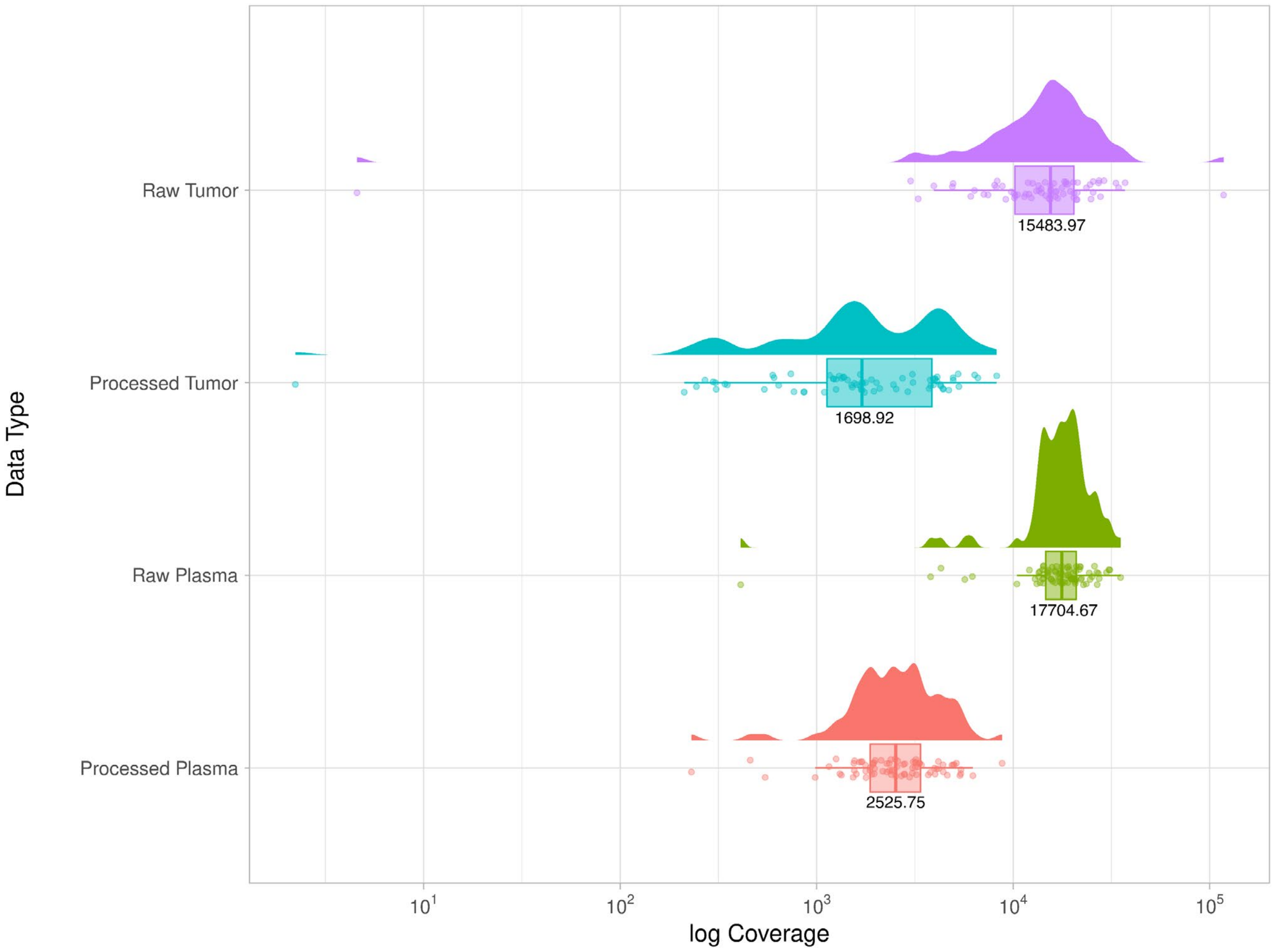

Figure S1. **Median sequencing coverage in tumor and plasma samples before and after bioinformatic processing.**

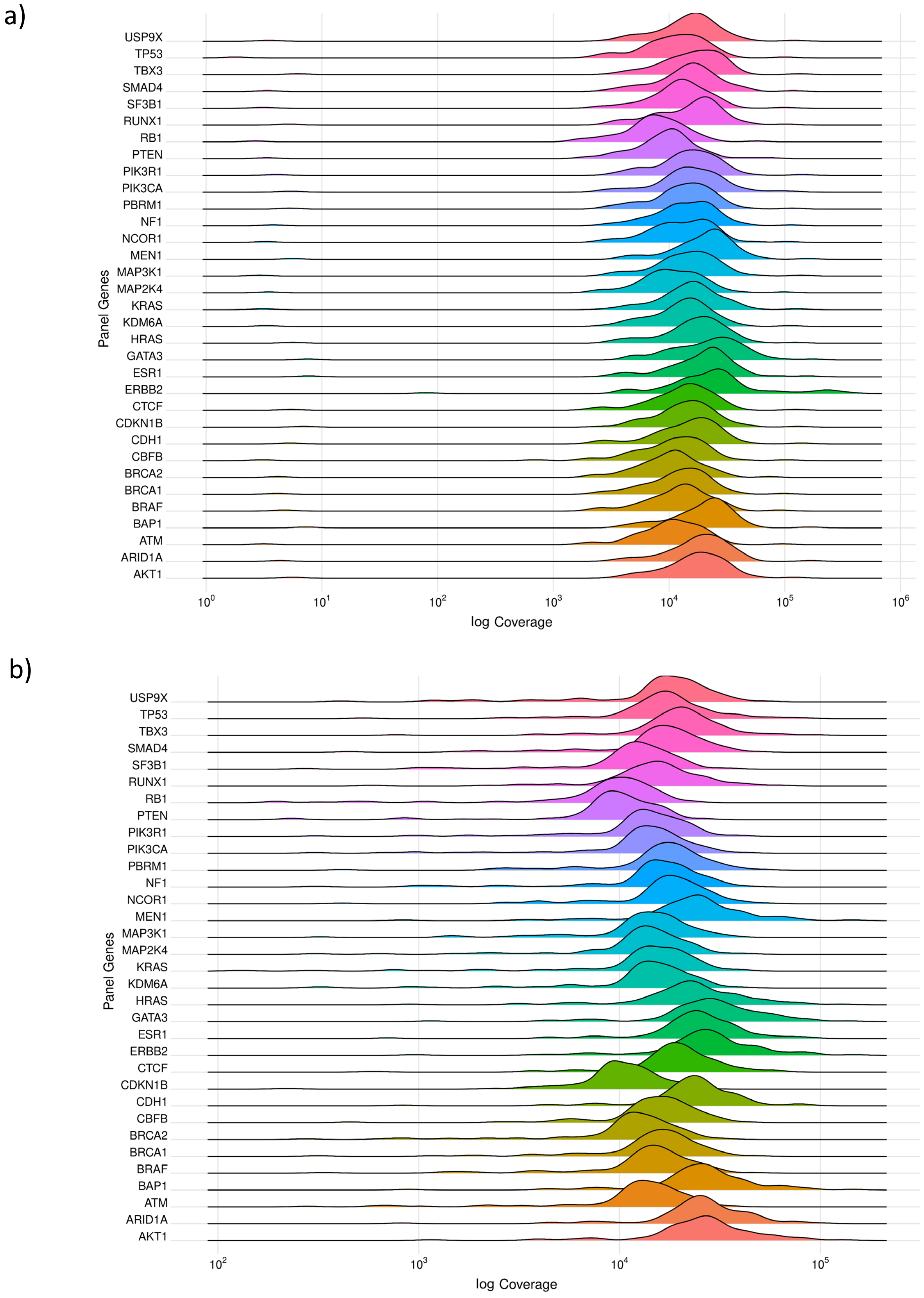

Figure S2. Sequencing coverage in a) tumor and b) plasma samples for the genes included in the custom panel

a)

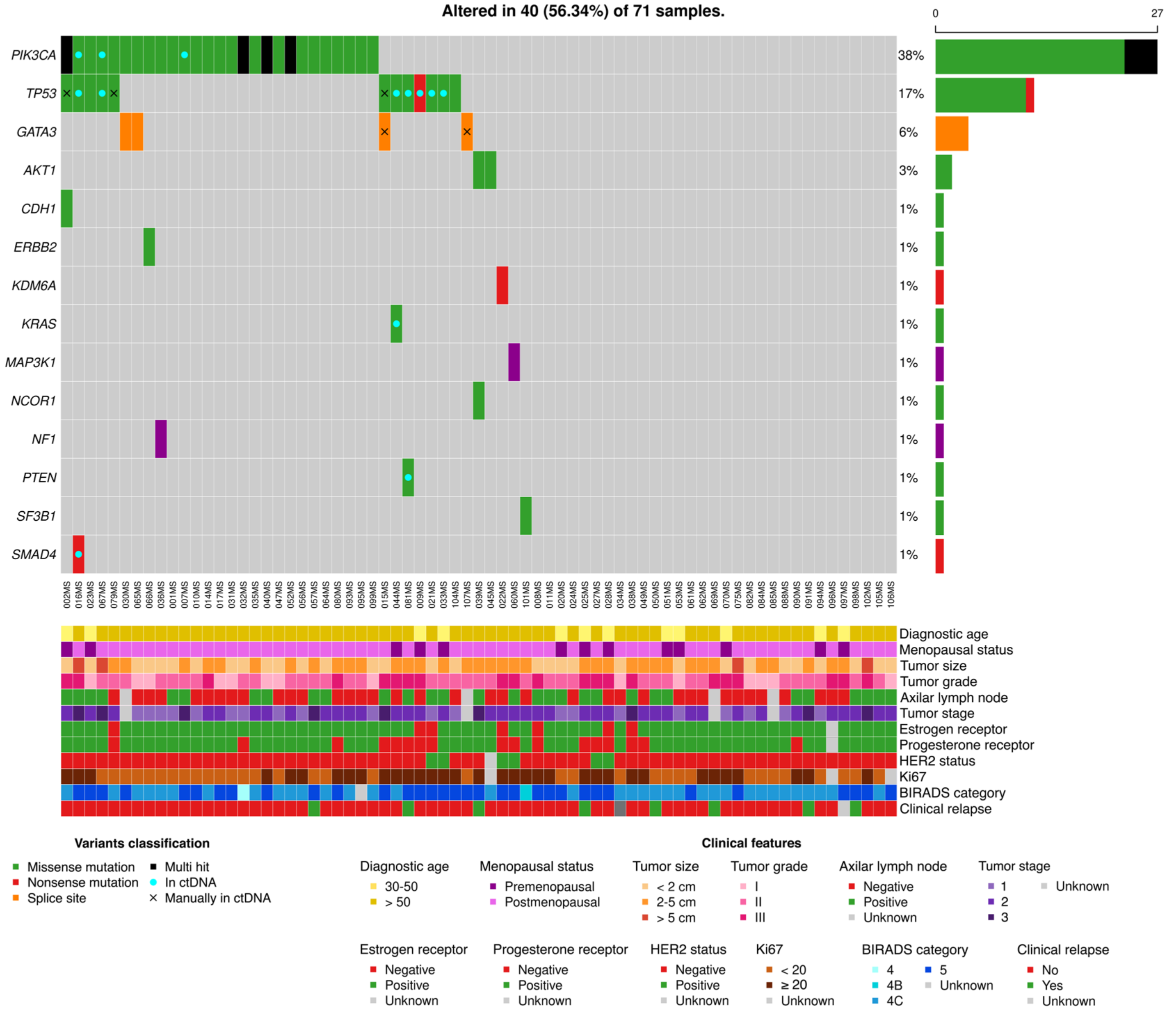

b)

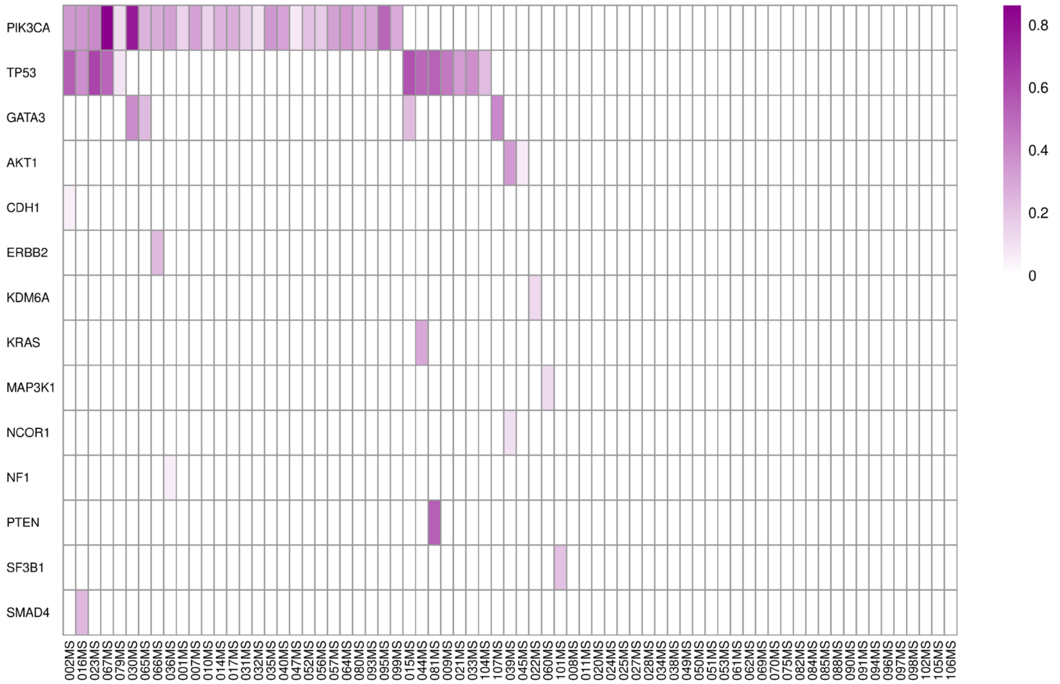

Figure S3. Mutations identified in the tumor DNA sequencing from the samples included in the study. a) Oncoplot showing in the *top part* the mutations observed in tumor samples indicating whether they were found in plasma samples. In the *bottom part,* the patients´ clinicopathological characteristics are represented. **b)** Variant allele frequencies of the mutations previously shown.

A)

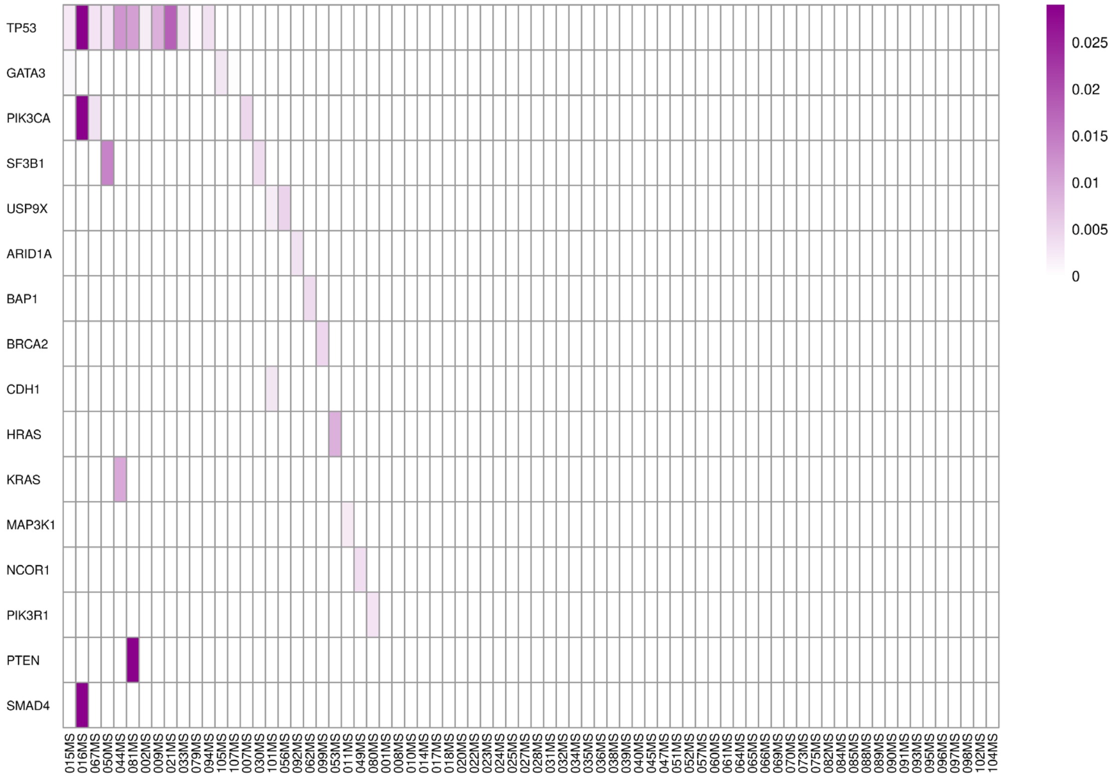

Figure S4. Variant allele frequencies of the identified plasma mutations.

a)

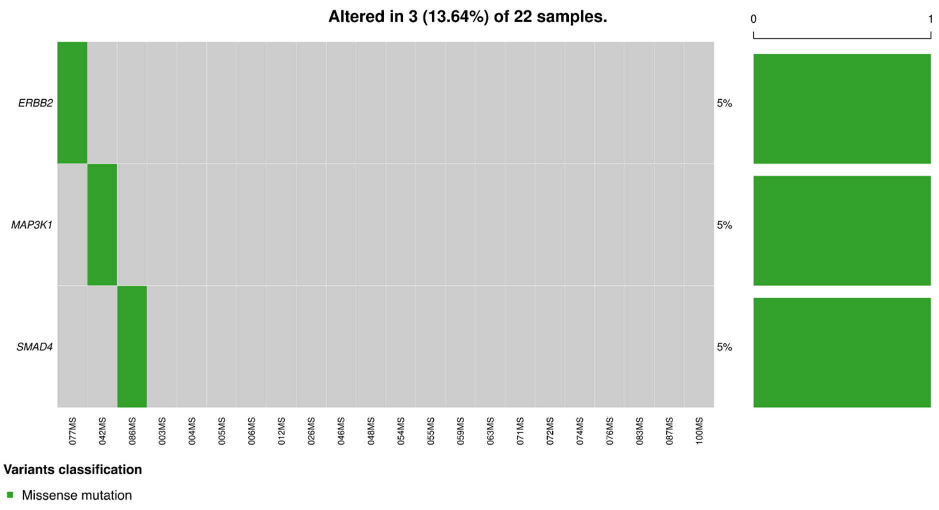

b)

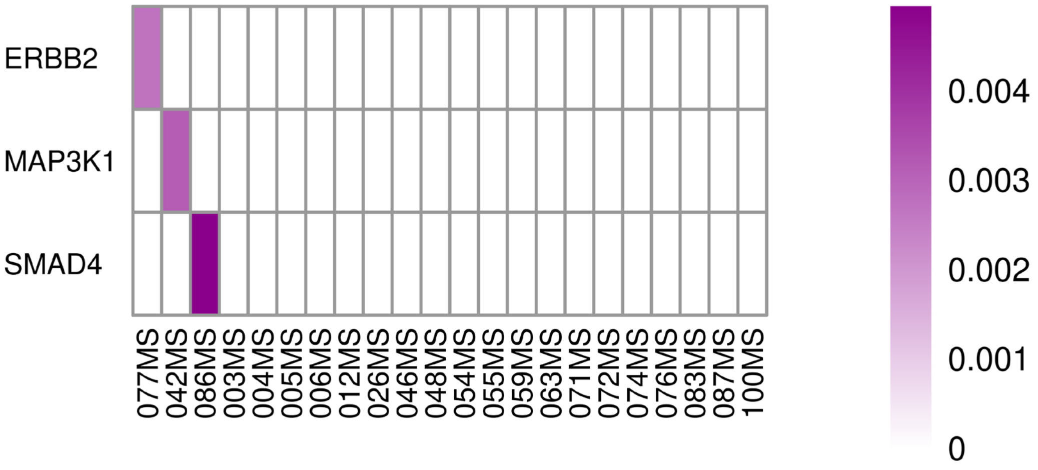

Figure S5. **Mutations identified in the plasma samples from healthy women. a)** Variants observed in the 22 sequenced plasma samples. **b)** Variant allele frequencies of the identified mutations.

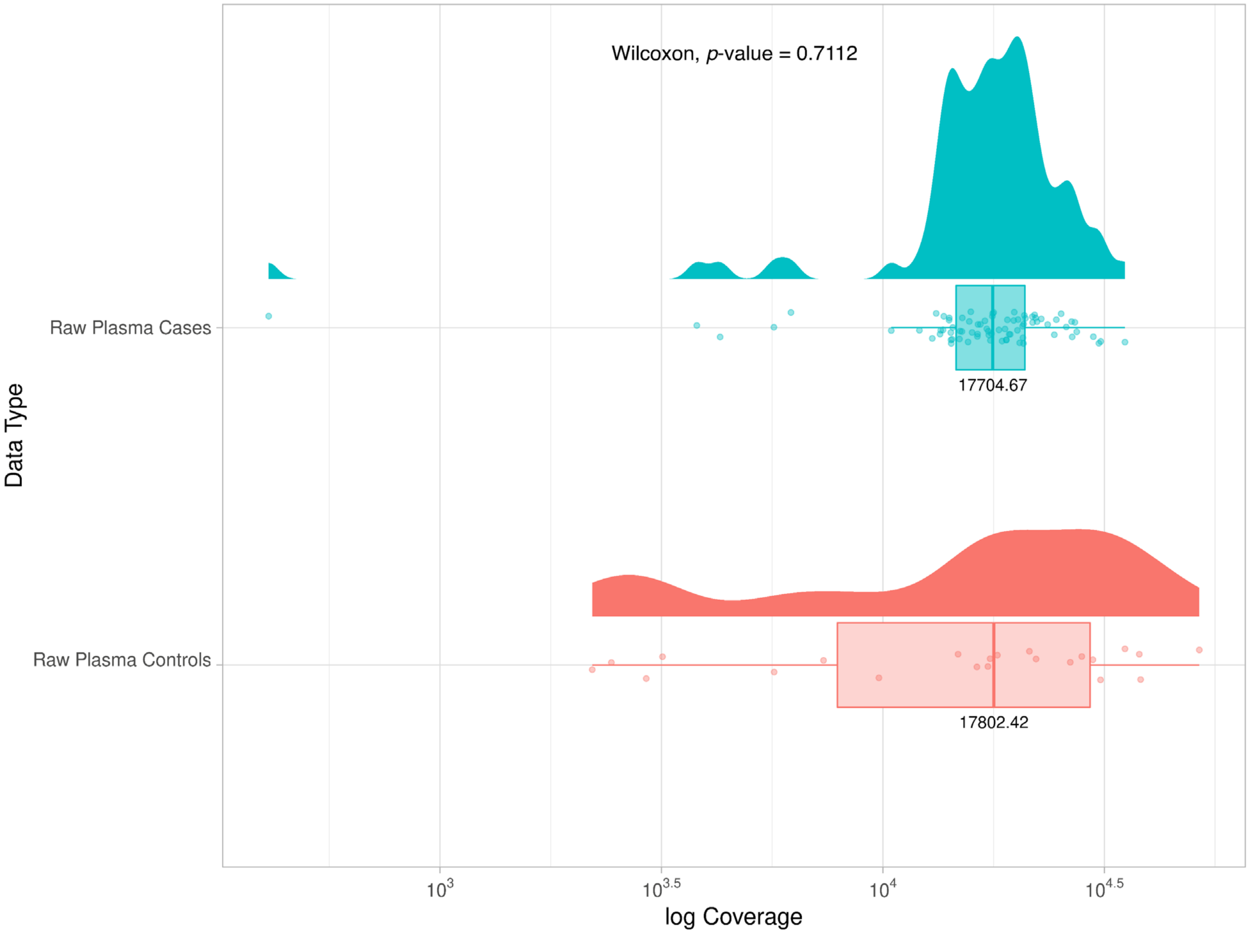

Figure S6. Median sequencing coverage in plasma samples from patients and healthy individuals. Not statistically significant Wilcoxon p-value test is shown.

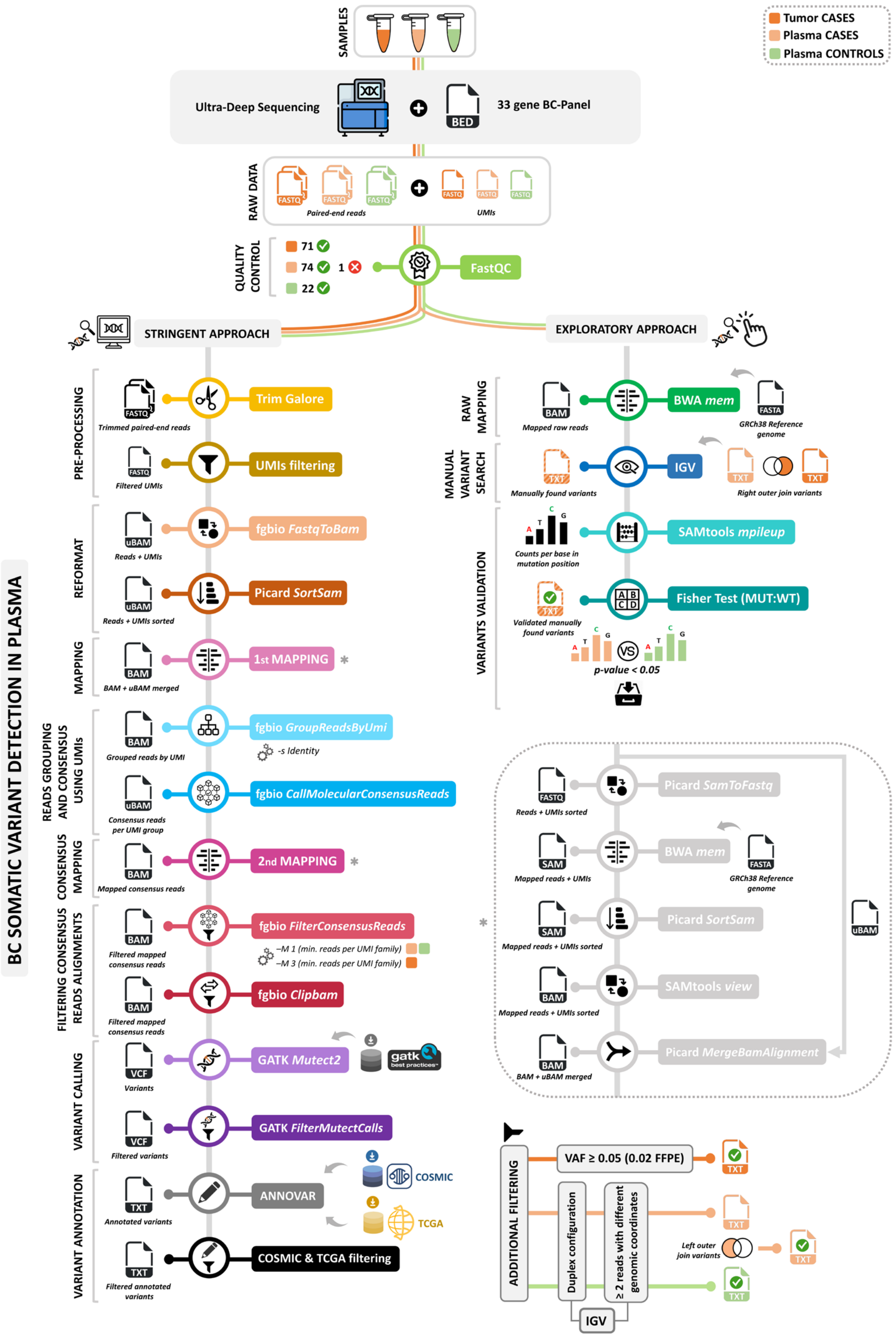

Figure S7. Schema representing the employed custom bioinformatic pipeline. Quality control was performed in raw sequencing data and the following steps were conducted: *Stringent approach*. Raw sequencing reads were (1) pre-processed and (2) reformatted for the subsequent (3) mapping step to the reference genome; (4) aligned reads were grouped by UMI and the generated consensus reads were (5) mapped again to the reference genome; (6) consensus reads supported by a minimum number of reads were kept and forward and reverse reads overlapping regions were removed; (7) variant calling using gatk resource bundle data for germline and non-cancer variants was performed followed by (8) variant annotation and selection. Tumor and plasma variants not detected in control samples and supported by more than 2 reads with different genomic coordinates and duplex configuration were identified. *Exploratory approach*. (1) Raw sequencing reads were mapped and (2) variants found called in tumor samples but not in plasma samples were manually checked and (3) assessed performing a Fisher test.
